# Lack of Tryptophan Hydroxylase-1 in Mice Results in Gait Abnormalities

**DOI:** 10.1371/journal.pone.0059032

**Published:** 2013-03-14

**Authors:** Georgette L. Suidan, Daniel Duerschmied, Gregory M. Dillon, Veronique Vanderhorst, Thomas G. Hampton, Siu Ling Wong, Jaymie R. Voorhees, Denisa D. Wagner

**Affiliations:** 1 Immune Disease Institute, Boston, Massachusetts, United States of America; 2 Program in Cellular and Molecular Medicine, Boston Children's Hospital, Boston, Massachusetts, United States of America; 3 Division of Hematology/Oncology, Boston Children's Hospital, Boston, Massachusetts, United States of America; 4 Department of Pediatrics, Harvard Medical School, Boston, Massachusetts, United States of America; 5 Department of Cardiology and Angiology I, University Heart Center Freiburg-Bad Krozingen, Freiburg, Germany; 6 Neurobehavior Laboratory, Harvard NeuroDiscovery Center, Boston, Massachusetts, United States of America; 7 Harvard Medical School, Boston, Massachusetts, United States of America; 8 Department of Neurology, Beth Israel Deaconess Medical Center, Boston, Massachusetts, United States of America; 9 Neuroscience Discovery Core, Framingham, Massachusetts, United States of America; National Cerebral and Cardiovascular Center, Japan

## Abstract

The role of peripheral serotonin in nervous system development is poorly understood. Tryptophan hydroxylase-1 (TPH1) is expressed by non-neuronal cells including enterochromaffin cells of the gut, mast cells and the pineal gland and is the rate-limiting enzyme involved in the biosynthesis of peripheral serotonin. Serotonin released into circulation is taken up by platelets via the serotonin transporter and stored in dense granules. It has been previously reported that mouse embryos removed from Tph1-deficient mothers present abnormal nervous system morphology. The goal of this study was to assess whether Tph1-deficiency results in behavioral abnormalities. We did not find any differences between Tph1-deficient and wild-type mice in general motor behavior as tested by rotarod, grip-strength test, open field and beam walk. However, here we report that *Tph1* (−/−) mice display altered gait dynamics and deficits in rearing behavior compared to wild-type (WT) suggesting that tryptophan hydroxylase-1 expression has an impact on the nervous system.

## Introduction

Tryptophan hydroxylase (TPH) is the rate-limiting enzyme involved in the biosynthesis of serotonin [1], [2]. Recently, it has been established that two forms of tryptophan hydroxylase exist: termed TPH1 and TPH2. Under normal conditions,TPH1 is predominantly expressed in a wide variety of non-neuronal cells such as enterochromaffin cells of the gut, mast cells and the pineal gland [3], [4], [5]. In the blood, serotonin is primarily stored in the dense granules of platelets which undergo endocytosis via the serotonin transporter [3]. TPH2 is expressed in neurons of the central, peripheral and enteric nervous systems [3], [4], [5]. In adult life, there appears to be no substantial overlap in the expression of the two TPH isoforms [6], [7]. However, there is evidence that TPH1 mRNA is present in the raphé nuclei during postnatal development where it may have an impact on the nervous system [8].

Genetic deletion of *Tph1* has been linked to nervous system abnormalities in E12.5 mouse embryos removed from *Tph1* (−/−) mothers [9]. Specifically, embryos from *Tph1* (−/−) mothers revealed gross abnormalities such as overall size reduction, altered morphology of the rhombencephale regions and neopallial cortex and decreased mitotic activity in ventricular zone and roof of the neopallial cortex. Defects were not found in *Tph1* (−/−) embryos from heterozygous or WT mothers indicating that maternal *Tph1* plays a role in development of the CNS. Another potential source of TPH1 during development is from the placenta where it is expressed as early as E10.5 and has been shown to contribute to 5-HT levels in the embryonic forebrain during early development [10]. While it is unknown if the CNS abnormalities in null and heterozygous embryos from *Tph1* (−/−) mothers continue into adulthood, there are studies indicating that altered 5-HT signaling, especially during early postnatal development, can lead to other disorders such as anxiety in the adult [11], [12], [13], [14], [15].

Several phenotypes have been reported in *Tph1* (−/−) mice such as mild anemia [16], [17], decreased neutrophil recruitment to inflammatory sites [17], diabetes, in particular during pregnancy [18], [19], and cardiopathy [4] which could potentially lead to behavioral deficits in offspring of *Tph1* (−/−) mice.

Interestingly, it has recently been reported in humans that maternal use of selective serotonin reuptake inhibitors (SSRIs), which prevent uptake of peripheral serotonin into neurons as well as platelets, is linked to a more pronounced decrease in growth of fetal head circumference when compared to untreated maternal depression [20]. It is unknown whether this difference leads to long-term behavioral changes in children of women taking SSRIs during pregnancy.

Taken together, these previous observations led us to hypothesize that *Tph1* (−/−) mice born from null mothers could have behavioral abnormalities in adulthood. In the current study, we show that *Tph1* (−/−) mice born from knockout mothers have altered gait dynamics throughout life and deficits in rearing behavior with age when compared to age-matched wild-type mice. These data suggest that TPH1 has an impact on nervous system development and possibly maintenance.

## Materials and Methods

### Mice

All animal experiments were approved by the Animal Care and Use Committee of the Immune Disease Institute and the Harvard Medical Area standing committee on animals (#04761). Adequate measures were taken to minimize pain and discomfort. C57BL/6J (WT) mice were purchased from Jackson Laboratory (Bar Harbor, ME, USA). *Tph1* (−/−) mice were a kind gift, received from M. Bader, Max-Delbruck-Center, Berlin, Germany and were on C57BL/6 background. To avoid genetic drift, *Tph1* (−/−) mice were routinely back-crossed to C57BL/6J mice a minimum of five times prior to experimentation and were continued to be back-crossed routinely. All mice used for the behavioral experiments were male, age- and weight-matched. All *Tph1* (−/−) mice used for experiments were bred from *Tph1* (−/−) pairs.

Rotarod, grip strength, open field, metabolic activity, attention performance and social novelty preference tests were performed at the NeuroBehavior Laboratory of the Harvard NeuroDiscovery Center. These paradigms were run in the respective order listed on the same groups of mice (2–3 month old mice termed “young” and 8–9 month old mice termed “aged”). The number of animals used for these studies was: WT young n = 10, *Tph1* (−/−) young n = 10, WT aged n = 10, *Tph1* (−/−) aged n = 8. All mice were single-housed and acclimated to the facility for two weeks prior to experimentation.

Gait dynamics and electrocardiogrpahy (Mouse Specifics Inc, Quincy, MA, USA), and beam walk were performed at the Immune Disease Institute in that order on group-housed mice at two ages (2–3 month old mice termed “young” and an aged group termed “>60 week old mice”) except for electrocardiography which was only performed on the >60 week old mice. The number of animals used for these studies was: WT young: n = 6 (gait), n = 8 (beam walk); *Tph1* (−/−) young: n = 8 (gait), n = 7 (beam walk); WT aged n = 8 (gait, electrocardiography, beam walk), *Tph1* (−/−) aged n = 10 (gait, electrocardiography and beam walk). Different cohorts of mice were used for gait and beam walk for the “young” groups whereas the same cohort of mice was used for the aged group. Mice were housed in the same facility for two weeks prior to experimentation. All experimentation was conducted during the light phase.

Fasting blood glucose level measurements were performed at the Boston Children's Hospital animal facility on age- and gender-matched young male (WT: n = 4; *Tph1* (−/−): n = 4) and female (WT: n = 6; *Tph1* (−/−): n = 5) mice that were group-housed.

### Rotarod Task

The rotarod apparatus consisted of a 3 cm in diameter, elevated rod that rotates at different speeds (Ugo Basile; Comerio VA, Italy) to assess sensorimotor ability in each mouse. One hour prior to testing, each mouse was habituated to the rotarod at a fixed speed and was placed back on the rod if it fell off within a 300 second time interval. For testing, the rod was accelerated from 4 to 40 rpm over 180 seconds and time to fall off the apparatus was recorded. Two trials were conducted and times were averaged for between group comparisons.

### Grip-Strength Test

The grip-strength test apparatus consisted of a grasping trapeze connected to a force transducer (Ugo Basile). Each mouse was held by the base of the tail and placed in front of the grasping trapeze. Once the mouse grasped the trapeze, it was slowly pulled back until the pulling force overcame the mouse's grip strength. The grip-strength meter expresses the grip force in grams. Each mouse was submitted to five trials that were separated by an inter-trial interval of 15 minutes. Final score represents the average of the five trials for each mouse.

### Open-Field Activity Test

The open field test was used to study general activity in mice confined to a novel arena. The apparatus used was a 27.9×27.9 cm Plexiglas arena with three 16-beam infrared arrays (Med Associates, St. Albans, VT). Mice were acclimated to the testing room for at least 30 minutes prior to testing. At the beginning of each session, mice were placed into the center of the open field and allowed to freely explore for 60 minutes. The total distance traveled (centimeters) and vertical beam breaks (an indication of rearing activity) were automatically recorded.

### Gait Dynamics

Gait dynamics were assessed using the DigiGait system (Mouse Specifics Inc, Quincy, MA, USA) in age-matched WT and *Tph1* (−/−) mice at 8 weeks (young) and >60 weeks (aged) of age as previously described [21], [22]. Briefly, digital images of the ventral plane of the mice were captured at 150 frames/sec for each mouse as it walked on a transparent treadmill belt. Young mice walked at a speed of 24 cm/sec whereas aged mice walked at 16 cm/sec. The speeds selected were based on published studies and the ability of the majority of the mice in each group to maintain a steady velocity during the short bout of walking(<1 minute). Software automatically determined the area of each paw as it advanced towards, established contact with, and retreated from the treadmill belt, providing gait signals for each of the four limbs. Numerous postural and kinematic indices were calculated from the images and gait signals. Gait metrics for each mouse were calculated from its individual ∼12 strides, and group means were calculated.

### Beam Walk

The beam walk test was set up as previously described [23]. Briefly, a 6 mm wide, 36-inch long beam was raised 1.5 feet above the surface. A black box was placed at one end to entice the mouse to cross the beam making a walking distance of 30 inches. Each mouse was placed on the opposite end of the beam and allowed to cross the beam to the black box. Bedding materials from the home cage of each mouse were placed in the black box as extra incentive for the mouse to cross the beam. The beam and box were cleaned with ethanol between each mouse. Mice were trained for 2 days, 2 times per day before testing. For the testing period, a video camera was located at the end of the beam opposite of the black box to give a view of the animal's feet on the beam. The number of times each mouse had a foot slip off of the beam was counted by a blinded observer.

### Attention performance testing

A recently developed method for testing visual discrimination learning and attention performance was performed [24] and the methods are briefly outlined below.

### Food restriction

Five days prior to the start of operant conditioning experiments, all subjects were placed on a food restriction diet. On the first day, baseline body weights were recorded and mice were individually placed into clean cages with access to water only. Food rations (regular chow) were calculated as a function of each individual's bodyweight loss/gain from the previous day and delivered daily to maintain a stable 80–85% of free feeding weight. During the 5-day period preceding training, mice were given fifteen 20 mg casein pellets (BioServ, Frenchtown, NJ, USA) daily, habituating the mice to the rewards. The food restriction procedure was maintained for the entire training and testing period.

### Visual Discrimination Learning Procedure

In this lever-press learning paradigm, mice were required to discriminate between reinforced and non-reinforced levers in order to obtain a food reward. Previous experiments have demonstrated that learning of the lever/reward association to be dependent on integrity of the hippocampus [24]. Training consisted of one daily session ending after either 80 trials or 45 minutes of session time had elapsed. Each trial began with the presentation of both levers accompanied by a cued stimulus light directly above one of the levers. Light-lever pairings were distributed in a random and counterbalanced manner across trials. Mice had to press the lever signaled by the light stimulus in order to obtain the food reward. The stimulus light remained illuminated until a lever-press was made or for a maximum duration of 30 seconds. Following a correct response, levers were retracted, a reward was delivered into the receptacle, and the receptacle light remained on until the pellet was retrieved (Med Associates).

### Attention procedure

Mixed-trial attention sessions followed an identical protocol to the discrimination procedure with the exception that the duration of the stimulus light presentation was reduced to 0.5, 1, 2, or 10 seconds. Regardless of the stimulus duration, subjects had 30 seconds to respond before an omission was recorded. Twenty trials at each stimulus duration were presented within a session (45 minutes maximum, 80 trials total), and stimulus durations and lever-stimulus pairings were distributed randomly and counterbalanced across trials.

### Preference for social novelty

A three-chambered rectangular box was used to evaluate sociability and preference for social novelty as previously described [25], [26]. Test mice were first placed in the middle chamber and allowed to explore all three chambers for ten minutes (habituation). After the habituation period, the test mouse was confined to the center chamber while an unfamiliar male mouse of the same genotype (stranger 1), that had no prior contact with the subject mouse, was confined to one of the side chambers in a small wire cage. The test mouse was then allowed to explore the entire social test box for a ten-minute session. At the end of the ten-minute session, the test mouse was confined to the center chamber while an unfamiliar mouse was confined in a small wire cage in the third chamber (stranger 2). The test mouse was then allowed to explore all three chambers to determine its preference for the new stranger. The amount of time spent in each chamber and the number of entries into each chamber were scored by an automated video-tracking system (TopScan; Cleversys, Reston, VA, USA). Data are represented as the time that the test mouse spent exploring stranger 1 divided by the total amount of time it was allowed to explore the chambers multiplied by 100% and the time that the test mouse spent exploring stranger 2 divided by the total amount of time it was allowed to explore the chambers multiplied by 100%.

### Metabolic/24-hour Activity Test

Monitoring animals in a familiar environment over an extended period of time is often necessary to detect impairments in circadian rhythms and/or metabolic activity. For that purpose we used the Comprehensive Lab Animal Monitoring System (CLAMS) from Columbus Instruments (Columbus, OH, USA). Mice were individually placed in CLAMS chambers (plastic cages, 20 cm×16 cm) for a period of three consecutive days (the first two days are necessary for familiarization to the chambers and behavioral measures were only analyzed during the final 24 hours). In these chambers, mice have free access to food (regular chow) and water.

### Measurement of blood glucose level

2–3 month old male and female *Tph1* (−/−) mice and their gender- and age-matched wild-type controls were fasted for 6 hours before measurement of blood glucose. Blood was obtained by tail-nick method and the glucose level was immediately determined using a glucometer (Clarity Advanced Blood Glucose Meter, Diagnostic Test Group, Boca Raton, FL, USA).

### Electrocardiography

Electrocardiograms were recorded non-invasively in awake *Tph1* (−/−) and WT mice, >60 weeks of age, as previously described using the ECGenie electrocardiography system (Mouse Specifics) [27]. Briefly, Einthoven signal-lead ECG signals were recorded passively from the underside of the mouse's paws as they rested on the instrumented platform. Data from ∼50 continuous ECG signals were used for analyses.

### Statistical Analysis

For vertical rearing counts, grip strength, and rotarod, a 2-way ANOVA was used to examine genotype and age effects as well as the interaction between these factors. For visual discrimination and open field habituation, a repeated measures ANOVA was added across session days and time respectively (Sigma Plot; San Jose, CA, USA). During attention testing, the percentage of correct responses was also separated by stimulus duration to provide an accurate measure of performance across varying degrees of attentional demand. When appropriate, Student-Newman-Keuls post hoc tests were used to compare statistical variation between groups. For beam walking, gait analysis, fasting blood glucose levels, heart rate and QRS times a two-tailed, unpaired student's t-test was performed between age groups due to the difference in treadmill speed in young and aged mice. Results were considered significant at P<0.05.

## Results

### Gait dynamics are altered in Tph1 (−/−) mice

Gait dynamics in *Tph1* (−/−) mice were significantly different when compared to age-matched controls. Young mice of both genotypes were made to walk on a treadmill at a speed of 24 cm/sec and >60 week old mice walking at 16 cm/sec. Stride length and swing duration were significantly decreased in the *Tph1* (−/−) mice (n = 6–10 per group. Young: P<0.001; >60 weeks: P<0.001 for stride length and swing duration). Consistent with decreased stride length, *Tph1* (−/−) mice of both age groups displayed increased stride frequencies (n = 6–10 per group. Young: P<0.001; >60 weeks: P = 0.002), indicating that they were taking more steps in the same period of time (Table 1).

**Table 1:** *Tph1* (−/−) mice demonstrate alterations in gait dynamics.

	C57BL/6J young	*Tph1* (−/−) young	P-value	C56BL/6 >60 weeks	*Tph1*(−/−) >60 weeks	P-value
**Swing(ms)**	96±2.0	85±2.0	<0.01	105±4.0	90.2±3.0	0.01
**Swing of stride (%)**	40.5±0.5	38.7±0.6	<0.05	33.3±0.8	32.3±1.1	n.s.
**Brake (ms)**	45±4.0	45±2.0	n.s.	62±5.0	80±3.0	<0.01
**Propel (ms)**	94±4.0	89±3.0	n.s.	147±7.0	114±5.0	<0.01
**Stance (ms)**	141±2.0	134±2.0	<0.05	209±4.0	194±5.0	<0.05
**Stance of stride (%)**	59.5±0.5	62.4±0.6	<0.05	66.7±0.8	67.7±1.1	n.s.
**Stride (ms)**	237±2.0	219±4.0	<0.01	315±7.0	286±5.0	<0.01
**Stride length (cm)**	5.7±0.15	5.3±0.1	<0.01	5.0±0.1	4.6±0.1	<0.01
**Brake of stance (%)**	33.6±2.6	34±1.3	n.s.	30.5±2.7	41.7±1.8	<0.01
**Propel of stance (%)**	66.4±2.6	66.1±1.3	n.s.	69.5±2.7	58.3±1.8	<0.01
**Stride frequency (steps/sec)**	4.3±0.0	4.7±0.1	<0.01	3.3±0.1	3.6±0.1	<0.01
**Paw angle (deg)**	10.4±0.9	9.9±1.2	n.s.	14.2±1.2	17.1±1.5	n.s.
**Paw angle variability (deg)**	18.8±1.2	20.5±1.5	n.s.	8.5±1.1	7.3±0.7	n.s.
**Paw Area at Peak Stance (mm^2^)**	485±39	501±34	n.s.	464±43	517±38	n.s.
**Stride length CV (CV%)**	14.6±0.7	17.5±1.1	<0.05	18.7±1.2	25.7±1.9	<0.01
**Paw Area Variability at Peak Stance (mm^2^)**	29±3.0	29±3.0	n.s.	32±3.0	52±8.0	0.05
**Speed of treadmill (cm/s)**	24	24		16	16	
**Number of animals**	6	8		8	10	

A stride is comprised of a swing duration (limb in air) and a stance duration (paw in contact with the treadmill belt). The stance is comprised of a brake duration and a propulsion duration. The paw angle is the outward angle that the paw makes relative to the long axis of the mouse during walking. Gait metrics were described previously [21], [45].

### Grip strength, rotarod and balance beam

**Figure 1:**
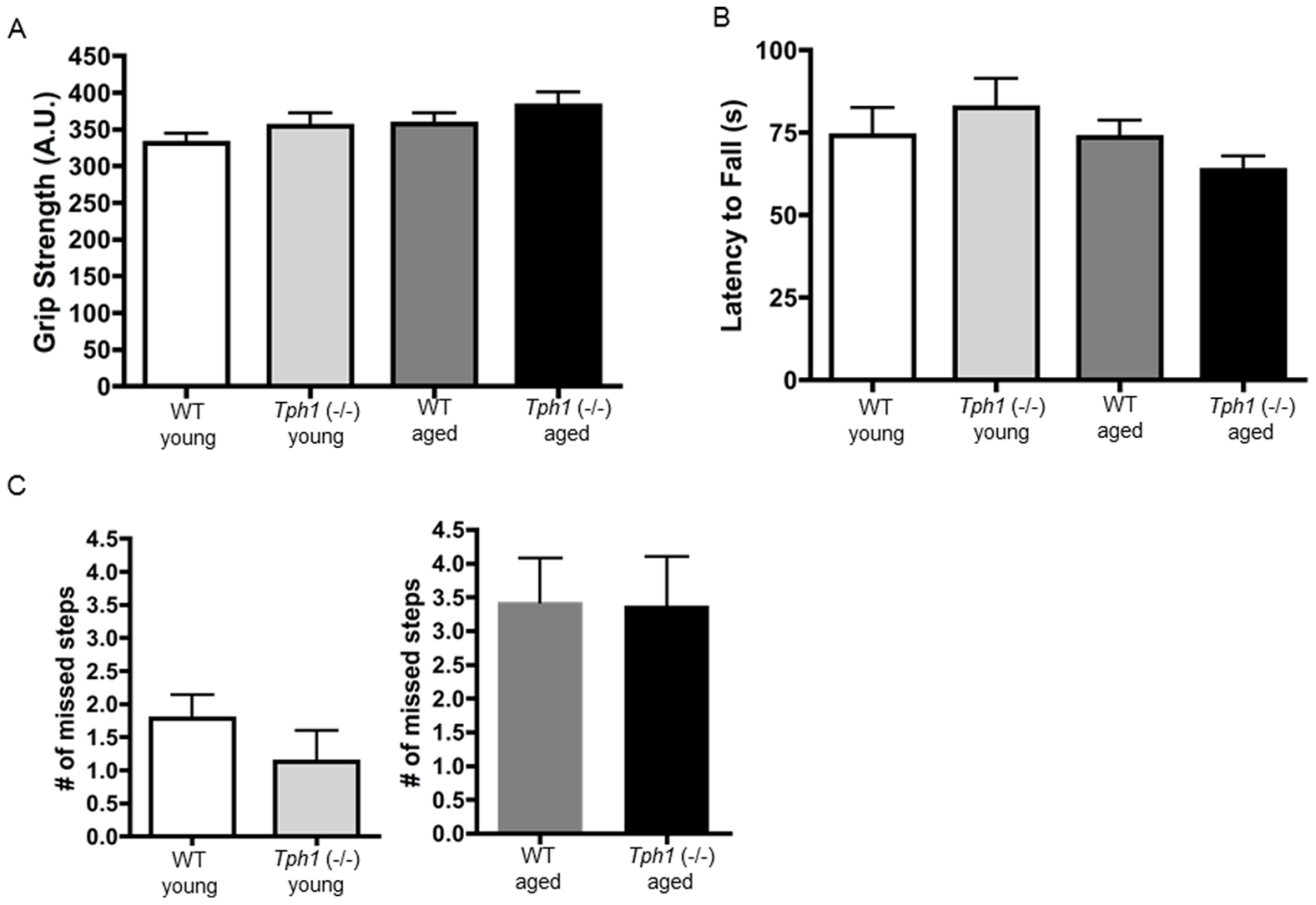
Muscular strength, balance, motor coordination and neuro-muscular integration are normal in *Tph1* (−/−) mice. Grip strength (A) and latency to fall off the rotarod apparatus (B) are similar in WT and *Tph1* (−/−) mice in both age groups. There were no significant differences in number of missed steps while crossing the beam (C) within age groups.

We did not observe an effect of age or genotype in muscle strength, motor coordination and/or neuro-muscular integration as tested by grip strength (genotype effect: F_(1,37)_ = 2.143; P = 0.152; Figure 1A) and rotarod performance (genotype effect: F_(1,37)_ = 0.009; P = 0.922; Figure 1B). To further ensure that *Tph1* (−/−) mice displayed normal locomotor function we utilized the beam walk test (Figure 1C). In this task, there were no differences in the number of hind leg slips between age-matched WT and *Tph1* (−/−) mice indicating that changes in gait dynamics were not due to problems with fine motor coordination and balance [23]. Additionally, fasting blood glucose levels were similar between young *Tph1* (−/−) and WT animals of both sexes (Male: P = 0.50; Female: P = 0.53) (Figure S1 A, B). Electrocardiogram results indicated that *Tph1* (−/−) and WT mice at >60 weeks of age had similar heart rate (P = 0.56) and QRS values (P = 0.32) indicating that the *Tph1* (−/−) mice have normal heart rhythm and ventricular conduction. (Figure S1 C, D).

Calorimetry experiments conducted over a 72 hr period demonstrated no differences in metabolic activity as assessed by oxygen consumption, carbon dioxide production and respiratory exchange rate between the WT and *Tph1* (−/−) mice of both ages (data not shown). Food intake and weight were also similar in WT and *Tph1* (−/−) mice of both ages (data not shown).

### Open field activity

**Figure 2:**
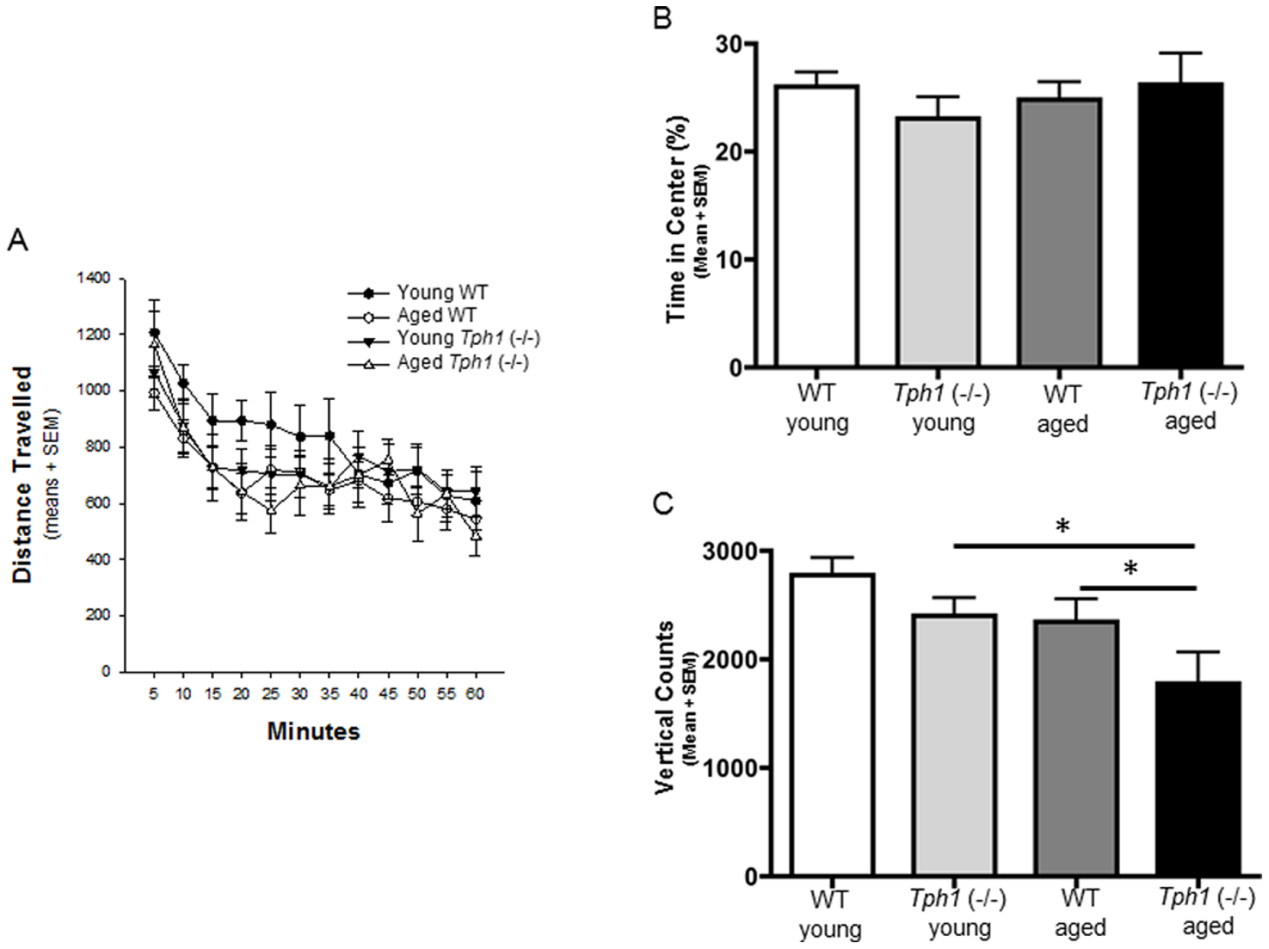
Aged *Tph1* (−/−) demonstrate significantly fewer vertical rearing counts compared to WT mice. Distance traveled in the open field chamber (A) and time spent in the center of the chamber (B) was similar between WT and *Tph1* (−/−) mice. Aged Tph1−/− mice have significantly fewer vertical counts as assessed by 2-way ANOVA; P<0.05 (C). Post hoc analysis revealed that there was a significant difference between aged WT and *Tph1* (−/−) (* P<0.05) and a significant difference between young and aged *Tph1* (−/−) mice (* P<0.05).

Spontaneous (total distance travelled) and motivated locomotion (rearing activity) were assessed in a single, 60-minute open field session. Our results indicate that all of the mice tested, regardless of age or genotype, demonstrated a significant habituation to the novel environment as reflected by a progressive decrease in activity across session time (F_(11,455)_ = 21.371; P<0.001). There was no effect of age or genotype on the total distance travelled (genotype effect: F_(1, 455)_ = 0.243; P = 0.625) (Figure 2A) or the percentage of time spent in the center (genotype effect: F_(1, 37)_ = 0.150; P = 0.701) (Figure 2B). However, an examination of the total number of vertical rearings, a measure of motivated activity and curiosity, demonstrated a significant difference in age (F_(1, 37)_ = 5.394; P = 0.027) and genotype (F_(1, 37)_ = 7.989; P = 0.008) (Figure 2C). These data demonstrate that a lack of peripheral serotonin may lead to deficits in motivated locomotion that progressively worsen with age.

### Visual Discrimination learning and attention performance

**Figure 3:**
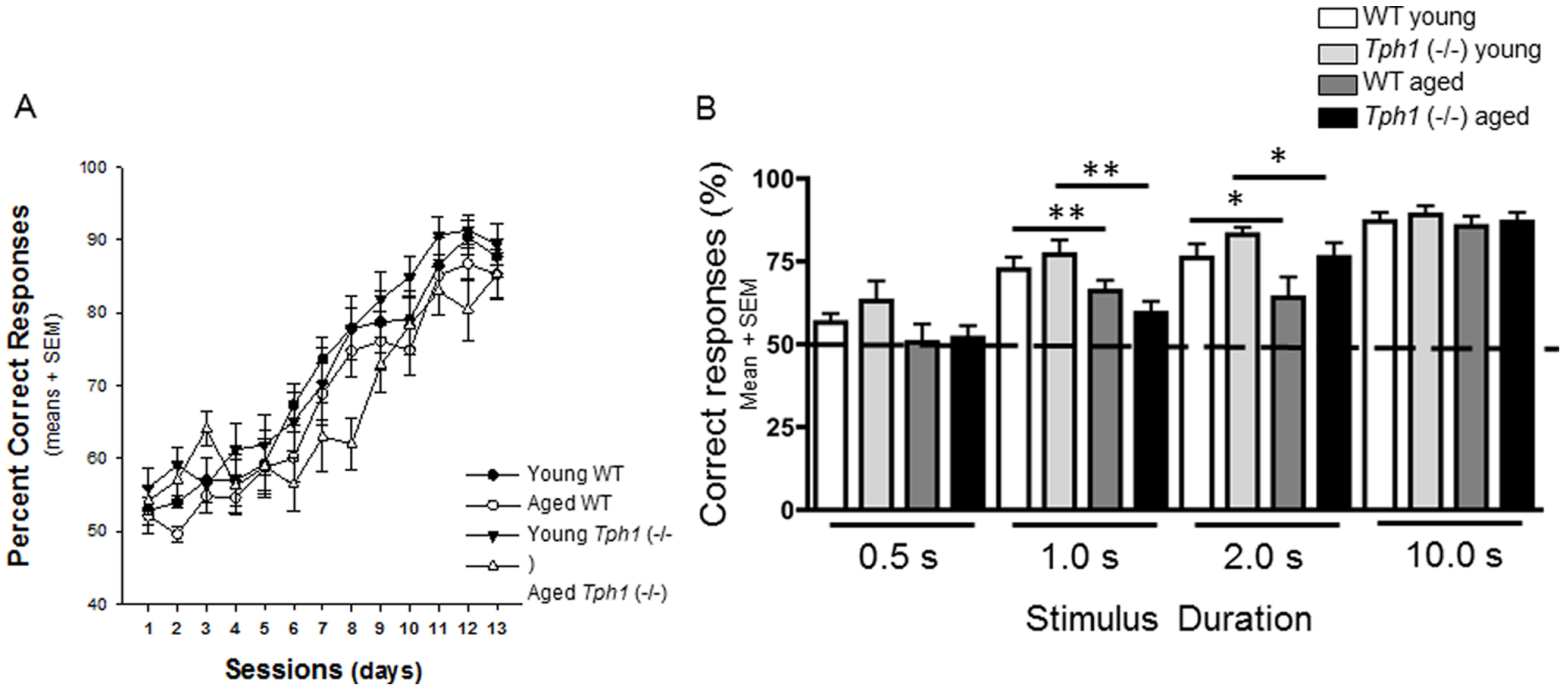
Lack of peripheral serotonin does not result in attention impairment. Ability to learn the association between the stimulus light and reward in WT and Tph1 (−/−) mice showed a strong trend indicating that aged Tph1 (−/−) mice may acquire this association more slowly, however, overtime they demonstrated successful learning across daily sessions (A). Overall, aged mice of both genotypes showed a significant deficit in performance during trials of 1 (**P<0.01) and 2 seconds (*P<0.05) (B).

Previous work has confirmed that learning the association between an illuminated lever with reward is dependent on the integrity of the hippocampal formation, while attention performance in the same task is dependent on the medial prefrontal cortex [24]. For this reason, the current paradigm allows us to examine cognitive abilities dependent on two separate brain structures using the same paradigm. Both WT and *Tph1* (−/−) mice were able to learn the association between the stimulus light and reward (session effect: F_(12,493)_ = 93.385; P<0.001) however there was a strong trend indicating that aged *Tph1* (−/−) mice may acquire this association slower (age×genotype: F _(36, 493)_ = 1.398; P = 0.06; Figure 3A). For attention testing, the amount of time the light remained illuminated above the lever was varied in order to alter attentional demand (the less time the light is on, the more attentional demand on the subject). As internal controls, the light was on for 0.5 seconds (performance indicated by chance 50%) and 10 seconds (an excess amount of time to make the correct choice, not dependent on attention). Our results indicate no effect of genotype at any of the current time points examined; however, age did cause a significant deficit in performance during trials of 1 (age effect: F_(1,37)_ = 10.047; p = 0.003) and 2 seconds. (age effect: F_(1,37)_ = 4.212; p = 0.048; Figure 3B). The results indicate that lack of peripheral serotonin did not cause severe cognitive alterations in adulthood on tasks examining both hippocampal and prefrontal cortex function.

### Preference for social novelty

**Figure 4:**
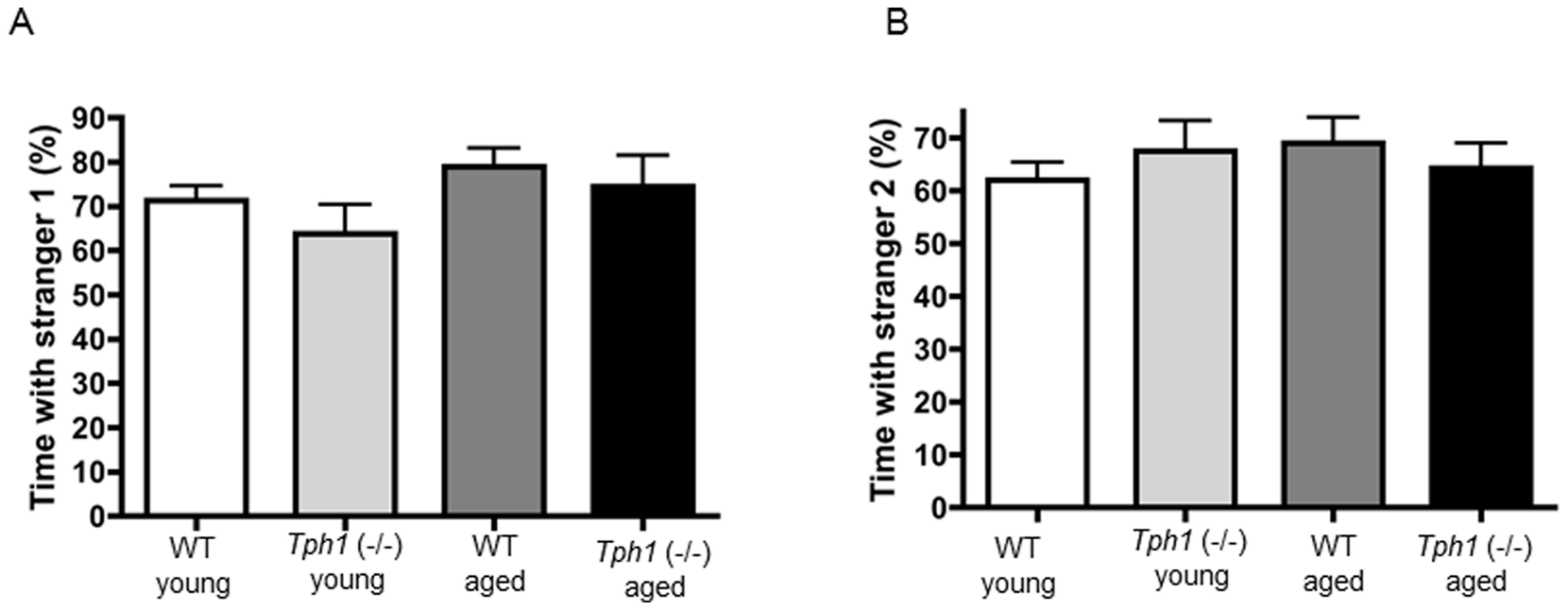
*Tph1* (−/−) mice have a normal response to social novelty. *Tph1* (−/−) mice demonstrate similar responses to a stranger mouse (A) and to a novel mouse (B). The percentage of time that the test mouse spent exploring stranger 1 was comparable to age-matched WT animals (A) and the time that the test mouse spent exploring stranger 2 was comparable to age-matched WT mice (B).

To determine the extent that Tph1-deficiency played a role in the mouse's preference for social novelty, we used a protocol previously described to assess autistic-like characteristics [25]. This procedure assesses both general sociability and the preference for novelty in mice. Our results show that *Tph1* (−/−) mice and age-matched controls demonstrate increased time spent with a stranger mouse compared to the empty chamber (Figure 4A) however, there was no effect of genotype (F_(1,37)_ = 1.243; P = 0.273) or age (F_(1,37)_ = 2.921; P = 0.097) on this performance. These results indicate that *Tph1* (−/−) mice demonstrate a preference for social interactions similar to controls. In addition, *Tph1* (−/−) mice and controls also displayed increased exploration of a second stranger mouse when compared to one previously investigated (Figure 4B) although again there was no effect of genotype (F_(1,37)_ = 0.007; P = 0.933) or age (F_(1,37)_ = 0.149; P = 0.702). This demonstrates both an intact memory for a co-specific odor and the preference for social novelty. Overall these data indicate that Tph1-deficiency does not lead to deficits in social behavior.

## Discussion

The goal of this study was to assess whether *Tph1* (−/−) offspring from *Tph1* (−/−) mothers results in behavioral abnormalities. The most striking behavioral differences that we report in these *Tph1* (−/−) mice are alterations in gait dynamics. Gait dynamics can be used to determine a subject's ability for balance, proprioception and coordination. In addition, these indices commonly reflect motor impairment in diseases such as Parkinson's disease, nerve injury and pain [28], [29], [30]. As gait dynamics can be altered due to changes in neural circuitries specific for locomotion, but also due to general motor and balance disturbances, we performed a battery of tests on these mice such as rotarod, grip strength, balance beam and open field tasks. Although we found altered gait dynamics in young and aged *Tph1* (−/−) mice compared to age-matched controls, we did not find any disturbances in general motor and balance abilities. However, in the open field arena aged *Tph1* (−/−) mice demonstrated a significant decrease in vertical rearings and a trend towards a decrease in rotarod performance as compared to WT mice of the same age. Vertical rearings have been used in some studies as a means of assessing motor abnormalities and motivated locomotion [31]. Because this effect was more pronounced with age, our data may indicate that the motor disturbances brought on by a lack of peripheral serotonin may be progressive. The current results are also consistent with previous reports indicating that at approximately 3 months of age, *Tph1* (−/−) mice demonstrate no deficits in basic locomotor activity [32]. However, it should also be noted that these studies were not conducted on older mice and these mice were derived on a mixed C57BL/6 x 129 SvJ background, whereas our mice were bred onto the C57BL/6J background.

Interestingly, the robust decrease in stride length found in the *Tph1* (−/−) is similar to that in a recently published C57BL/6J mouse model of Parkinson's disease using 1-methyl-4-phenyl-1,2,3,6-tetrahydropyridine to induce lesions of the substantia nigra [28]. Morelli et al., moreover, recently showed that mice deficient in the serotonin transporter (5-HTT−/−) also exhibited decreased stride length, and these studies were conducted using mice on the 129S6 background [33]. This genetic deletion not only affected neuronal serotonin uptake, but also uptake of peripheral serotonin in platelets making the mice deficient in platelet serotonin similar to the currently examined *Tph1* (−/−) mice. These mice also exhibited deficiencies in beam walk and rotarod whereas the *Tph1* (−/−) mice only displayed deficits in gait.

The finding of significantly decreased swing duration in both young and aged Tph1−/− animals compared to WT is intriguing. In contrast to stride length and stance duration, which change in a speed dependent manner and are modified by primary afferent sensory feedback, there is little variation in swing duration [34]. Studies using neonatal brainstem spinal preparations have demonstrated that serotonin modulates rhythmic locomotor-like activity both when applied exogenously to the spinal cord [35] or via endogenous release [36] involving 5HT7 and 5HT2A receptors [37]. Questions remain whether structural changes occur in the serotonergic-raphe spinal pathways of *Tph1* (−/−) during development, and/or during aging. Further studies are necessary to identify whether changes in these systems underlie the decreased swing duration in the *Tph1* (−/−) mice.

Recent clinical studies have shown that mutations of the *Tph1* gene rendering it less active are correlated with an increased prevalence of ADHD in children by 1.5–2.5 fold, suggesting that ADHD may be related to the lack of exposure to maternal serotonin during gestation [38]. As these mutations decrease the amount of maternal serotonin that the offspring are exposed to during gestation, we hypothesized that Tph1−/− mice born from knockout mothers could also develop a similar phenotype including attention deficits. To test this, we utilized the recently described 2-choice visual discrimination and attention test [24]. Although our results demonstrate a strong trend that aged *Tph1* (−/−) mice may acquire the association between light and reward at a slower rate (p = 0.06), each group demonstrated successful learning across daily sessions. This result demonstrates that hippocampal function in *Tph1* (−/−) is preserved. This finding is consistent with previous studies showing no changes in learning, memory or motivation in offspring of mothers in which the SSRI paroxetine (Paxil) was administered during gestation [39]. In addition, *Tph1* (−/−) mice showed no overt deficits in attention performance as indicated by performance at the 1 and 2 second stimulus durations. However, it should be noted that in the current study there was a significant decrease in attention performance between 2 to 8 months of age. This overall decrease in performance at 8 months of age may have made it difficult to determine an effect of genotype.

Previous results show that *Tph1* (−/−) mice on the C57BL/6 background have no abnormalities in anxiety-related behaviors tested in either the elevated plus maze or hold board task [2]. Deficits in social interaction are important early markers for autism and related neurodevelopmental disorders, and previous studies have shown social abnormalities in mice with attention deficits [40]. Furthermore, disorders involving social interaction in humans have been linked to abnormalities in serotonergic systems [41], [42] and it is established that autistic children have abnormal gait dynamics such as decreased stride length [43]. Therefore, we tested *Tph1* (−/−) mice in a model of social novelty. We found no differences in preference for social novelty in *Tph1* (−/−) mice compared to WT mice. It has been shown that anxiety/compulsive behavior and depression-related disorder testing were normal in *Tph1* (−/−) mice [32].

As mentioned earlier, several abnormalities have been reported in *Tph1* (−/−) mice such as diabetes [18], [19], decreased cardiac function under anesthesia [4] and anemia [16], [17] that could potentially contribute to behavioral differences. Studies performed on *Tph1* (−/−) mice from our colony demonstrate that the mice used for this work do not have increased blood glucose levels after 6-hour fasting. It is possible that differences in background strains account for this discrepancy. We also did not find any differences between >60 week old *Tph1* (−/−) and WT mice in heart rate and QRS interval duration indicating that these mice have normal rhythm and ventricular depolarization, thus it is unlikely that cardiac dysfunction plays a role in gait and rearing differences. Mice used for these studies were not anesthetized during ECG recordings. We have recently confirmed in our *Tph1* (−/−) colony that these mice have mild anemia, as previously reported [16], [17] however, it is unlikely to affect their gait.

Here, we report gait alterations and reduced motivated locomotion in murine *Tph1* (−/−) offspring born from knockout mothers. As previous studies did not find developmental CNS abnormalities in *Tph1* (−/−) mice from *Tph1* (+/−) and *Tph1* (+/+) mothers, we speculate that the behavioral differences in these mice are mainly due to CNS abnormalities induced by lack of maternal TPH1 exposure during gestation. In utero use of SSRIs is a very understudied field even though these antidepressants are commonly prescribed during pregnancy [44]. Our data support the few studies showing that there may be adverse behavioral affects associated with their use. As SSRI use is also known to prevent platelet uptake of peripheral serotonin, there could be a link between platelet storage of serotonin and behavioral abnormalities that warrants further investigation.

## Supporting Information

Figure S1 ***Tph1*** **(−/−) mice had normal fasting blood glucose levels (A, B), heart rate (C) and QRS interval duration (D).**
(TIF)
