# Correction: Lack of Tryptophan Hydroxylase-1 in Mice Results in Gait Abnormalities

**DOI:** 10.1371/annotation/49097405-57c1-4923-875a-9216de91d230

**Published:** 2013-08-16

**Authors:** Georgette L. Suidan, Daniel Duerschmied, Gregory M. Dillon, Veronique Vanderhorst, Thomas G. Hampton, Siu Ling Wong, Jaymie R. Voorhees, Denisa D. Wagner

A funding organization was incorrectly omitted from the Funding Statement. The first sentence of the Funding Statement should read: "This work was generously supported by National Heart, Lung and Blood Institute of the National Institutes of Health grant R01 HL041002 (to D.D.W.) and a National Research Service Award from the National Institutes of Neurological Disease and Stroke of the National Institutes of Health grant 1F32NS073245 (to G.L.S.) and supported in part by the Harvard NeuroDiscovery Center." 

