# Supplementary figures and images for: Lack of Tryptophan Hydroxylase-1 in Mice Results in Gait Abnormalities
Source: PLoS One. 2013 Mar 14;8(3):e59032. doi: 10.1371/journal.pone.0059032 (PMC3597584; doi:10.1371/journal.pone.0059032)

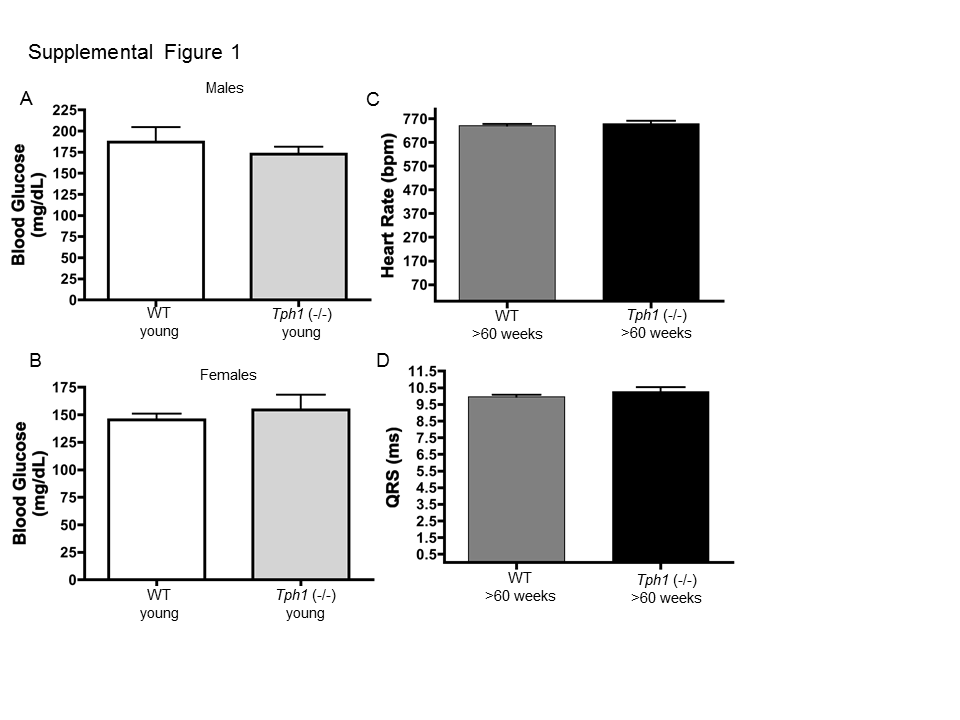

Supplement: Figure S1 — Tph1 (−/−) mice had normal fasting blood glucose levels (A, B), heart rate (C) and QRS interval duration (D). (TIF) [file pone.0059032.s001.tif]
